# Trends in Screen Time Use Among Children During the COVID-19 Pandemic, July 2019 Through August 2021

**DOI:** 10.1001/jamanetworkopen.2022.56157

**Published:** 2023-02-15

**Authors:** Monique M. Hedderson, Traci A. Bekelman, Mingyi Li, Emily A. Knapp, Meredith Palmore, Yanan Dong, Amy J. Elliott, Chloe Friedman, Maren Galarce, Diane Gilbert-Diamond, Deborah Glueck, Christine W. Hockett, Maristella Lucchini, Julia McDonald, Katherine Sauder, Yeyi Zhu, Margaret R. Karagas, Dana Dabelea, Assiamira Ferrara

**Affiliations:** 1 Kaiser Permanente Northern California Division of Research, Oakland; 2 Lifecourse Epidemiology of Adiposity and Diabetes (LEAD) Center, University of Colorado Anschutz Medical Campus, Aurora; 3 Department of Epidemiology, Johns Hopkins Bloomberg School of Public Health, Baltimore, Maryland; 4 Avera Research Institute, Sioux Falls, South Dakota; 5 Department of Pediatrics, University of South Dakota School of Medicine, Sioux Falls; 6 Department of Epidemiology, Geisel School of Medicine at Dartmouth, Hanover, New Hampshire; 7 Department of Medicine, Geisel School of Medicine at Dartmouth, Hanover, New Hampshire; 8 Department of Pediatrics, Geisel School of Medicine at Dartmouth, Hanover, New Hampshire; 9 Department of Pediatrics, University of Colorado School of Medicine, Aurora; 10 Department of Psychiatry, Columbia University Irving Medical Center, New York, New York

## Abstract

**Importance:**

The COVID-19 pandemic led to widespread lockdowns and school closures that may have affected screen time among children. Although restrictions were strongest early in the pandemic, it is unclear how screen time changed as the pandemic progressed.

**Objective:**

To evaluate change in children’s screen time from before the pandemic to during the pandemic, from July 2019 through August 2021.

**Design, Setting, and Participants:**

This is a longitudinal cohort study with repeated measures of screen time collected before the pandemic and during 2 pandemic periods. Children aged 4 to 12 years and their parent were enrolled in 3 pediatric cohorts across 3 states in the US participating in the Environmental Influences of Child Health Outcomes (ECHO) Program. Data analysis was performed from November 2021 to July 2022.

**Exposures:**

COVID-19 pandemic period: prepandemic (July 2019 to March 2020), pandemic period 1 (December 2020 to April 2021), and pandemic period 2 (May 2021 to August 2021).

**Main Outcomes and Measures:**

The primary outcomes were total, educational (not including remote school), and recreational screen time assessed via the ECHO Child Media Use questionnaire. Linear mixed-effects models were used for screen time adjusted for child’s age, number of siblings, sex, race, ethnicity, and maternal education.

**Results:**

The cohort included 228 children (prepandemic mean [SD] age, 7.0 [2.7] years; 100 female [43.9%]) with screen time measured during the prepandemic period and at least once during the pandemic period. Prepandemic mean (SD) total screen time was 4.4 (3.9) hours per day and increased 1.75 hours per day (95% CI, 1.18-2.31 hours per day) in the first pandemic period and 1.11 hours per day (95% CI, 0.49-1.72 hours per day) in the second pandemic period, in adjusted models. Prepandemic mean (SD) recreational screen time was 4.0 (3.5) hours per day and increased 0.89 hours per day (95% CI, 0.39-1.39 hours per day) in the first pandemic period and 0.70 hours per day (95% CI, 0.16-1.25 hours per day) in the second pandemic period. Prepandemic mean (SD) educational screen time was 0.5 (1.2) hours per day (median [IQR], 0.0 [0.0-0.4] hours per day) and increased 0.93 hours per day (95% CI, 0.67-1.19 hours per day) in the first pandemic period and 0.46 hours per day (95% CI, 0.18-0.74 hours per day) in the second pandemic period.

**Conclusions and Relevance:**

These findings suggest that screen time among children increased during the COVID-19 pandemic and remained elevated even after many public health precautions were lifted. The long-term association of increased screen time during the COVID-19 pandemic with children’s health needs to be determined.

## Key Points

**Question:**

Did children’s screen time change from before to during the COVID-19 pandemic?

**Findings:**

In this cohort study of 228 children aged 4 to 12 years, prepandemic mean total screen time increased 1.75 hours per day in the first pandemic period and 1.11 hours per day in the second pandemic period.

**Meaning:**

These findings suggest that screen time among children increased during the COVID-19 pandemic and remained elevated after many public health precautions were lifted.

## Teaser

This cohort study evaluates changes in children’s screen time from before the pandemic to during the pandemic, from July 2019 through August 2021.

## Introduction

Excessive screen time among children is associated with obesity-promoting health behaviors (ie, increased sedentary behavior and decreased sleep) and adverse mental health (eg, depressive symptoms, anxiety, internalizing and externalizing behaviors, and increased craving behaviors).^1^ The COVID-19 pandemic initially led to widespread school closures, shelter-in-place laws, closure of recreational facilities and sports, increases in parents working from home, and social distancing recommendations, all of which may have impacted screen time among children. Increases in screen time during the early phase of the pandemic may have been inevitable as many parents transitioned to work from home and children attended online school. Screen time during the early pandemic period may have been beneficial by allowing children to connect with friends and learn new things outside of school during periods of strict social distancing and remote learning.^2^ However, it is possible that once these behaviors are adopted, they will be hard to reverse, and whether the pandemic resulted in long-term changes in children’s screen time remains unclear.

Most of the existing studies on screen time were cross-sectional and examined screen time among different cohorts of people before and during the pandemic and were conducted outside the US.^3^ The few prospective studies to date^4,5,6,7,8,9^ were restricted to the early stage of the pandemic. Therefore, it remains unclear what happened to children’s screen time as the pandemic persisted and what types of screen time were most affected.

The aim of this study was to test the hypothesis that screen time increased during the COVID-19 pandemic by examining changes in screen time from the prepandemic period (July 1, 2019, to March 1, 2020) to pandemic period 1 (December 1, 2020, to April 30, 2021) and pandemic period 2 (May 1 to August 31, 2021). We leveraged longitudinal data collected via the Environmental Influences on Child Health Outcomes (ECHO) Program at these 3 time points and in 3 states across the US with varying degrees of pandemic restrictions. We explored sociodemographic correlates of screen time during the study period.

## Methods

### Study Design and Population

This longitudinal cohort study followed a subset of caregiver-child dyads in ECHO. The ECHO program is a collaboration between 69 established pediatric cohort studies collecting new data under a common protocol since 2019 to explore the association of early life environmental exposures with children’s health outcomes.^10^ Single and cohort-specific institutional review boards monitored human subject activities and the centralized ECHO Data Analysis Center. All participants in this study provided written informed consent. This manuscript follows the Strengthening the Reporting of Observational Studies in Epidemiology (STROBE) reporting guideline for cohort studies.

**Figure 1.**
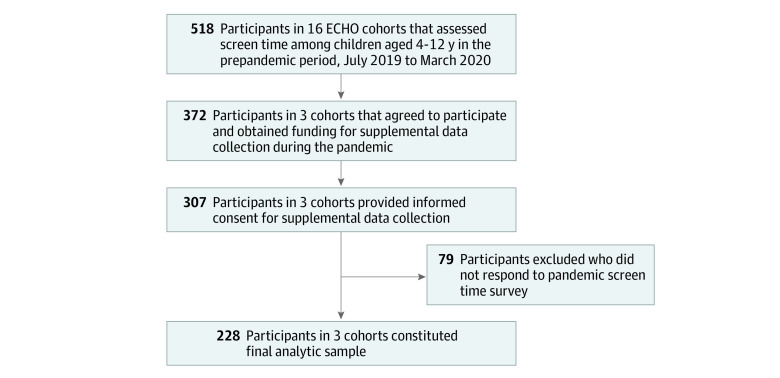
Flowchart of Inclusion Criteria for Analytic Cohort in the Environmental Influences of Child Health Outcomes (ECHO) Program

Of the 16 ECHO cohorts with prepandemic measures of screen time data, 3 agreed to participate and obtained funding for supplemental data collection during the pandemic (Figure 1 and eTable 1 in Supplement 1). Parents of children aged 4 to 12 years old who had screen time measures during the prepandemic period were contacted. Participants were recruited by phone, email, or text, and assessments were performed in person at a research visit or remotely by the parent or caregiver proxy report. Participants provided written informed consent and were included in the analytic sample if at least 1 follow-up measure during either of the 2 pandemic periods was completed.

### Measures

**Figure 2.**
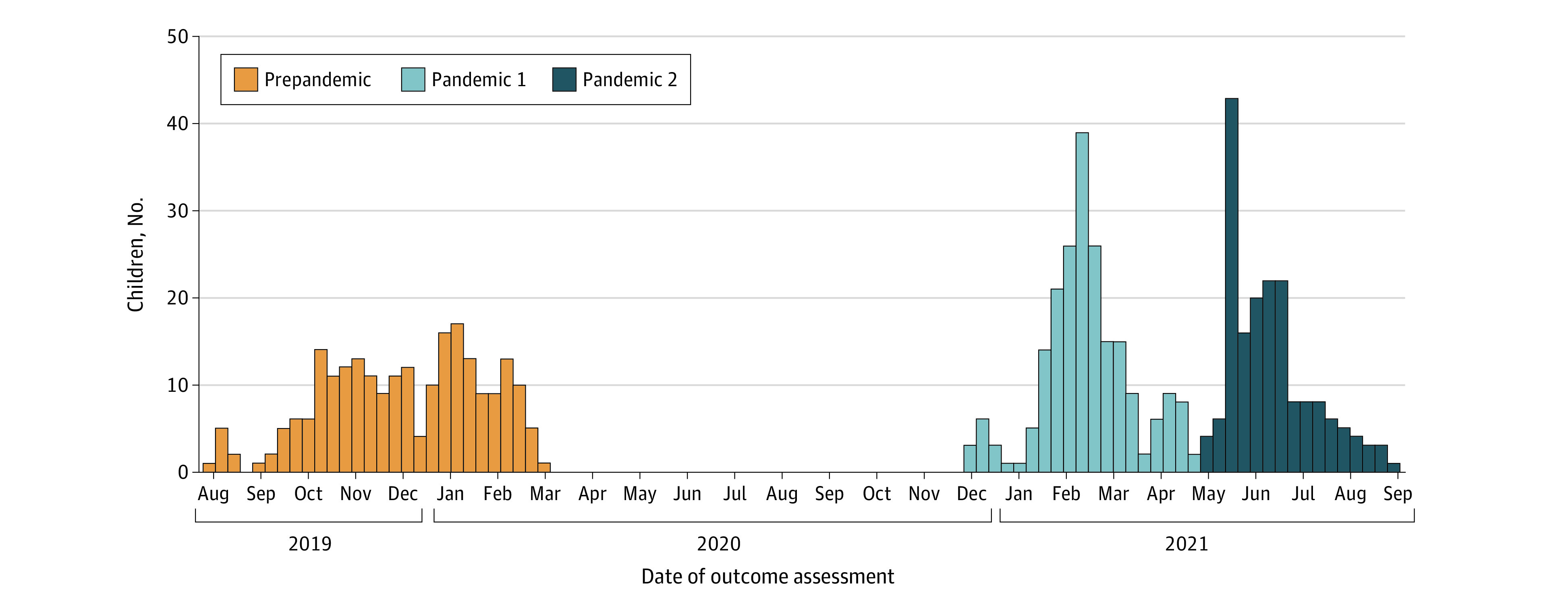
Date of Screen Time Outcome Assessment Graph shows number of children assessed for screen time each month between July 2019 and August 2021.

Children’s screen time measures were collected during the 8-month period before the COVID-19 pandemic (July 1, 2019, to March 1, 2020) as part of the ECHO protocol. Repeated measures of the same behaviors were collected during 2 pandemic periods: December 1, 2020, to April 30, 2021, and May 1 to August 31, 2021, from the same dyads (Figure 2).

Screen time was assessed with the ECHO Child Media Use questionnaire, which was modified from the Common Sense Census.^11^ Type and frequency of TV, tablet, computer, and smartphone use was reported separately for weekdays and weekends by the parent or caregiver. We calculated a weighted average of weekday and weekend duration for each screen time activity and created 3 categories of screen time activity: total, educational, and recreational. Total screen time comprised time spent on 10 specific activities: watching TV shows, DVDs, or videos; playing video games; playing games on an education game device; playing computer games; playing games or using apps on a smartphone or tablet; video chatting; doing homework on a computer or tablet; browsing websites; doing anything else on a computer; and doing anything else on a smartphone or tablet. Playing games on an educational game device and doing homework on a computer or tablet were included in educational screen time; however, because this survey was developed before the pandemic, screen time for remote schooling was not included. All other activities were considered recreational screen time. The child’s social media access was assessed with the following question answered by the parent or caregiver: “Does the child have one or more of his/her own social media accounts (for example, Facebook, Twitter, Snapchat, Instagram)?” Measures of activities performed together with a caregiver were assessed and dichotomized as some of the time or most of the time and never or hardly ever.

We assessed sociodemographic characteristics via self-report. Categorical sociodemographic variables include child’s sex assigned at birth (male or female), child’s age and race and ethnicity (Hispanic all races, non-Hispanic Black, non-Hispanic other [ie, Asian, American Indian or Alaska Native, and multiracial], and non-Hispanic White race), highest level of maternal education (high school or general education development or less, some college, bachelor’s degree, or master’s degree or higher), and annual household income (<$30 000, $30 000-$49 999, $50 000-$74 999, $75 000-$99 999, or ≥$100 000). We also assessed whether COVID-19 affected the mother’s employment and work modalities. Race and ethnicity were assessed in this study as imperfect measures of other social determinants of health and should not be interpreted as biological variables.

### Statistical Analysis

Data analysis was performed from November 2021 to July 2022. We summarized frequencies and percentages of sociodemographic characteristics of participants and provided descriptive summaries (mean and SD) of screen time measures for the 3 data collection periods. We performed linear mixed-effects models (R package lme4) for continuous outcomes, including weighted average screen time duration, weighted average educational screen time duration, and weighted average recreational screen time duration.^12^ All models included time period of data collection as the exposure of interest and were adjusted for child’s age, sex, race, ethnicity, number of siblings, and maternal education. Random intercepts for cohorts and participants were specified in models to account for within-cohort correlation and repeated outcome measures from the same child. A secondary analysis was performed with the outcome of change in screen time between the first or second pandemic time period and the prepandemic period, adjusting for the aforementioned covariates and the child’s baseline (prepandemic) measure of screen time. All data analyses were performed in R statistical software version 4.1.0 (R Project for Statistical Computing). Statistical significance was defined as α = .05.

## Results

Of 317 participants who consented to supplemental data collection, there were 228 children from 3 cohorts (Colorado, South Dakota, and California) included in the analysis (overall response rate, 71.9%). See eTable 2 in Supplement 1 for the comparison of demographic characteristics of responders and nonresponders. Children were a mean (SD) of 7.0 (2.7) years old at the time of their prepandemic screen time measure, and 100 (43.9%) were female. Sixty-five children (28.5%) were Hispanic, 18 (7.9%) were non-Hispanic Black, 37 (16.2%) were non-Hispanic other, and 108 (47.4%) were non-Hispanic White. One hundred fifty-seven (68.9%) of these children’s mothers had a bachelor’s degree or higher (Table 1). Compared with the Colorado and South Dakota cohorts, children from the California cohort were younger, more likely to be Hispanic, and to have lower levels of maternal education (eTable 3 in Supplement 1).

**Table 1.** Characteristics of Children Aged 4-12 Years Who Participated in the Screen Time Study Within the Environmental Influences of Child Health Outcomes Program

Characteristic	Children, No. (%) (N = 228)^a^
Sex	
Female	100 (43.9)
Male	128 (56.1)
Baseline child age, mean (SD), y	7.0 (2.7)
Baseline age group, y	
4 to <5	115 (50.4)
5 to <9	50 (21.9)
≥9	63 (27.6)
Race and ethnicity	
Hispanic all races	65 (28.5)
Non-Hispanic	
Black	18 (7.9)
White	108 (47.4)
Other^b^	37 (16.2)
State of study cohorts	
California	115 (50.4)
Colorado	74 (32.5)
South Dakota	39 (17.1)
Maternal education^c^	
Some college, no degree or lower	71 (31.1)
Bachelor’s degree	80 (35.1)
Master’s degree or higher	77 (33.8)
Annual household income, $	
<30 000	11 (4.8)
30 000-49 999	24 (10.5)
50 000-74 999	32 (14.0)
75 000-99 999	32 (14.0)
≥100 000	124 (54.4)
Missing	5 (2.2)
No. of siblings in household	
0	15 (6.6)
1	101 (44.3)
2	65 (28.5)
3	29 (12.7)
≥4	13 (5.7)
Missing	5 (2.2)
How COVID-19 affected maternal job (% yes)	
Moved to working remotely or from home	89 (39.0)
Missing	<5^d^
Did not have a paying job before COVID-19	23 (10.1)
Missing	<5^d^
Lost the job permanently or temporarily or reduced work hours	56 (24.6)
Missing	<5^d^
Got a new job or increased work hours	29 (12.7)
Missing	<5^d^

^a^ Children were included in this sample (n = 228) if they had at least one of the same screen time outcomes for both prepandemic (July 1, 2019, to March 15, 2020) and 1 pandemic period (December 1, 2020, to April 30, 2021, or May 1 to August 31, 2021). There were 211 children (92.5%) with a measure during the first pandemic time point and 179 (78.5%) with a measure in the second pandemic time period. A total of 162 children (71.1%) had measures at both pandemic time points.

^b^ Non-Hispanic other includes 13 Asian, 7 American Indian or Alaska Native, and 17 multiple race children.

^c^ Some college, no degree, or lower includes associate degree, trade school, high school degree, general education development or equivalent, and less than high school diploma. Master’s degree or higher also includes (professional) doctoral degree.

^d^ Percentages were not computed for cell sizes of <5.

Table 2 shows the means and SDs of the screen time measures at each of the 3 data collection periods. Overall, the mean total screen time per day, mean recreational screen time per day, and mean education screen time per day increased from the prepandemic period to pandemic period 1 and remained elevated at pandemic period 2 (Table 2). During the prepandemic period, 10 children (4.4%) reported having at least 1 social media account, whereas 20 (9.5%) and 20 (11.2%) reported having accounts at pandemic periods 1 and 2, respectively. Thirty-three children (14.5%) watched TV with caregivers at the prepandemic period, 32 children (15.2%) did so during pandemic period 1, and 35 children (19.6%) did at pandemic period 2. One hundred eighty-three children (80.3%) played computer games with a caregiver at the prepandemic period and this increased to 85.3% (180 children) in pandemic period 1 and 84.4% (151 children) in pandemic period 2. One hundred seventy-five children (76.8%) played educational games with a caregiver in the prepandemic period, and that increased to 83.4% (176 children) in pandemic period 1 and 83.2% (149 children) in pandemic period 2. However, video games were less likely to be played with a caregiver during the pandemic (72.4% [165 children] prepandemic vs 129 children [61.1%] in pandemic period 1 and 64.2% [115 children] in pandemic period 2). In the Colorado and South Dakota cohorts, children’s reported total screen time were the longest prepandemic durations (5.2 hours and 4.9 hours, respectively), whereas prepandemic screen time for children in the California cohort was only 3.6 hours (eTable 4 in Supplement 1). Adjusted analyses found increases in total screen time, educational screen time, and recreational screen time from prepandemic to the pandemic data collection time periods (Table 3).

**Table 2.** Change in Screen Time Between Prepandemic and Pandemic Periods Among Children Aged 4-12 Years in the Environmental Influences of Child Health Outcomes Program

Outcomes	Prepandemic, July 1, 2019-March 15, 2020 (n = 228)	Pandemic 1, December 1, 2020-April 30, 2021 (n = 211)	Pandemic 2 May 1-August 31, 2021 (n = 179)
Screen time, h/d			
Total duration^a^			
Mean (SD)	4.4 (3.9)	6.6 (4.5)	6.1 (4.5)
Median (IQR)	3.2 (1.9-5.3)	5.4 (3.5-8.7)	5.0 (3.0-7.5)
Weekday duration			
Mean (SD)	4.0 (4.2)	6.5 (4.7)	6.0 (4.7)
Median (IQR)	5.0 (3.0-7.5)	5.1 (3.0-8.5)	5.0 (2.6-7.6)
Weekend duration			
Mean (SD)	5.1 (3.7)	6.5 (4.3)	6.0 (4.2)
Median (IQR)	4.0 (2.8-6.5)	5.6 (3.6-8.2)	5.6 (3.6-8.2)
Educational duration^b^			
Mean (SD)	0.5 (1.2)	1.5 (2.0)	1.0 (1.5)
Median (IQR)	0.0 (0.0-0.4)	0.9 (0.1-2.1)	0.9 (0.1-2.1)
Recreational duration^c^			
Mean (SD)	4.0 (3.5)	5.3 (3.9)	5.2 (4.2)
Median (IQR)	2.9 (1.6-5.1)	4.1 (2.8-6.5)	4.1 (2.6-6.1)
Watching TV			
Mean (SD)	2.1 (1.9)	2.4 (1.7)	2.4 (2.3)
Median (IQR)	1.6 (1.0-2.7)	2.0 (1.2-3.0)	2.0 (1.1-2.8)
Playing games^d^			
Mean (SD)	1.3 (2.1)	2.0 (2.0)	2.0 (2.5)
Median (IQR)	0.6 (0.1-1.5)	1.3 (0.5-2.9)	1.3 (0.6-2.4)
Video chatting			
Mean (SD)	0.1 (0.3)	0.2 (0.5)	0.2 (0.7)
Median (IQR)	0.0 (0.0-0.0)	0.0 (0.0-0.0)	0.0 (0.0-0.2)
Browsing website^e^			
Mean (SD)	0.1 (0.3)	0.1 (0.4)	0.2 (1.0)
Median (IQR)	0.0 (0.0-0.0)	0.0 (0.0-0.0)	0.0 (0.0-0.0)
Others			
Mean (SD)	0.4 (1.1)	0.6 (1.1)	0.9 (2.4)
Median (IQR)	0.0 (0.0-0.4)	0.1 (0.0-0.7)	0.1 (0.0-0.9)
Social media account, No. (%) of children			
Yes	10 (4.4)	20 (9.5)	20 (11.2)
No	214 (93.9)	188 (89.1)	157 (87.7)
Missing	<5^f^	<5^f^	<5^f^
Watch TV with caregivers, No. (%) of children			
Some of the time or most of the time	33 (14.5)	32 (15.2)	35 (19.6)
Never or hardly ever	195 (85.5)	177 (83.9)	143 (79.9)
Missing	0	<5^f^	<5^f^
Play video game with caregivers, No. (%) of children			
Some of the time or most of the time	165 (72.4)	129 (61.1)	115 (64.2)
Never or hardly ever	56 (24.6)	77 (36.5)	60 (33.5)
Missing	7 (3.1)	5 (2.4)	<5^f^
Play computer game with caregivers, No. (%) of children			
Some of the time or most of the time	183 (80.3)	180 (85.3)	151 (84.4)
Never or hardly ever	37 (16.2)	26 (12.3)	20 (11.2)
Missing	8 (3.5)	5 (2.4)	8 (4.5)
Play games on an education game device with caregivers, No. (%) of children			
Some of the time or most of the time	175 (76.8)	176 (83.4)	149 (83.2)
Never or hardly ever	42 (18.4)	27 (12.8)	21 (11.7)
Missing	11 (4.8)	8 (3.8)	9 (5.0)
Play games or use apps on a smartphone or tablet with caregivers, No. (%) of children			
Some of the time or most of the time	118 (51.8)	120 (56.9)	112 (62.6)
Never or hardly ever	108 (47.4)	84 (39.8)	61 (34.1)
Missing	<5^f^	7 (3.3)	6 (3.4)
Video-chat with caregivers, No. (%) of children			
Some of the time or most of the time	161 (70.6)	137 (64.9)	125 (69.8)
Never or hardly ever	59 (25.9)	67 (31.8)	48 (26.8)
Missing	8 (3.5)	7 (3.3)	6 (3.4)

^a^ Includes playing games on an education game device (eg, Leapster/LeapPad, LeapFrog Epic, Playtime Pad, or V-Tech device, V-Smile, Mobigo, or Innotab) and doing homework on a computer or tablet. This is a weighted average of weekday and weekend duration.

^b^ Includes watching TV shows, DVDs, or videos, playing video games, playing computer games, playing games or using apps on a smartphone or tablet, video chatting, browsing websites, doing anything else on a computer, and doing anything else on a smartphone or tablet. This is a weighted average of weekday and weekend duration.

^c^ Includes watching TV shows, DVDs, or videos (include time spent watching on a TV set, computer, smartphone, and tablet; this includes watching shows that may have been previously recorded). This is a weighted average of weekday and weekend duration.

^d^ Includes playing video games, playing computer games, and playing games or using apps on a smartphone or tablet. This is a weighted average of weekday and weekend duration.

^e^ Includes doing anything else on a computer (eg, looking at pictures, looking up things, social networking, emailing, shopping, and text messaging) and doing anything else on a smartphone or tablet (eg, taking or looking at pictures, looking up things, social networking, emailing, text messaging, or using other types of apps not already covered). This is a weighted average of weekday and weekend duration.

^f^ Percentages were not calculated for cell sizes <5.

**Table 3.** Estimated Associations Between Total Screen Time, Educational Screen Time, and Recreational Screen Time and Pandemic Periods

Variable	Screen time, β (95% CI), h/d^a^		
	Total	Educational	Recreational
No. of observations	618	618	618
Time point^b^			
Pandemic 1	1.75 (1.18 to 2.31)	0.93 (0.67 to 1.19)	0.89 (0.39 to 1.39)
Pandemic 2	1.11 (0.49 to 1.72)	0.46 (0.18 to 0.74)	0.70 (0.16 to 1.25)
Age, y	0.51 (0.36 to 0.67)	0.09 (0.04 to 0.15)	0.48 (0.33 to 0.62)
No. of siblings	−0.35 (−1.16 to 0.43)	−0.20 (−0.47 to 0.07)	−0.25 (−0.02 to 0.47)
Male sex	0.01 (−0.36 to 0.39)	0.05 (−0.08 to 0.18)	−0.01 (−0.37 to 0.34)
Race and ethnicity			
Hispanic all races	2.61 (1.56 to 3.66)	0.62 (0.25 to 0.98)	2.21 (1.23 to 3.20)
Non-Hispanic			
Black	5.43 (3.87 to 6.98)	0.99 (0.44 to 1.54)	4.73 (3.28 to 6.19)
White	1 [Reference]	1 [Reference]	1 [Reference]
Other^c^	1.83 (0.67 to 2.98)	0.95 (0.55 to 1.35)	1.03 (−0.05 to 2.12)
Maternal education			
Some college, no degree or lower	1 [Reference]	1 [Reference]	1 [Reference]
Bachelor’s degree	−1.48 (−2.48 to −0.48)	−0.34 (−0.69 to 0.01)	−1.27 (−2.21 to −0.33)
Master’s degree or higher	−1.85 (−2.88 to −0.82)	−0.49 (−0.85 to −0.14)	−1.46 (−2.43 to −0.50)

^a^ Beta coefficients were calculated from linear mixed-effects models that include cohort and child as random intercepts to adjust for the within cohort and within child correlations. Each screen time outcome represents the weighted average of weekday and weekend parent-reported screen time. Maximum likelihood method was used to fit model. The analysis was conducted using the R package lme4.

^b^ The reference group is the prepandemic period, July 1, 2019, to March 15, 2020. Pandemic data collection period 1 is December 1, 2020, to April 30, 2021, and pandemic period 2 is May 1 to August 31, 2021.

^c^ Other includes Asian, American Indian or Alaska Native, and multiple races.

### Total Screen Time

Prepandemic mean (SD) total screen time was 4.4 (3.9) hours per day. Children had an increase in mean total screen time of 1.75 hours per day (95% CI, 1.18-2.31 hours per day) during the first pandemic period and 1.11 hours per day (95% CI, 0.49-1.72 hours per day) during the second pandemic period, compared with the prepandemic period. Throughout the study period, children who reported being members of self-identified racial or ethnic minoritized groups had more total screen time compared with non-Hispanic White children. Non-Hispanic Black children had 5.43 hours per day more (95% CI, 3.87-6.98 hours per day), Hispanic children had 2.61 hours per day more (95% CI, 1.56-3.66 hours per day), and non-Hispanic other race children had 1.83 hours per day more (95% CI, 0.67-2.98 hours per day) (Table 3).

### Educational Screen Time

Prepandemic mean (SD) educational screen time was 0.5 (1.2) hours per day (median [IQR], 0.0 [0.0-0.4] hours per day). Educational screen time increased 0.93 hours per day (95% CI, 0.67-1.19 hours per day) in the first pandemic period and 0.46 hours per day (95% CI, 0.18-0.74 hours per day) in the second pandemic period. Educational screen time was also longer among all racial or ethnic minoritized children compared with non-Hispanic White children: 0.99 hours per day (95% CI, 0.44-1.54 hours per day) longer among Black children, 0.62 hours per day (95% CI, 0.25-0.98 hours per day) longer among Hispanic all races children, and 0.95 hours per day (95% CI, 0.55-1.35 hours per day) longer among other racial and ethnic groups (Table 3).

### Recreational Screen Time

Prepandemic mean (SD) recreational screen time was 4.0 (3.5) hours per day. Recreational screen time increased 0.89 hours per day (95% CI, 0.39 to 1.39 hours per day) at the first pandemic period and 0.70 hours per day (95% CI, 0.16 to 1.25 hours per day) at the second pandemic period, compared with the prepandemic period. Racial and ethnic minoritized children also had longer durations of recreation screen time compared with non-Hispanic White children: 4.73 hours per day (95% CI, 3.28 to 6.19 hours per day) longer among Black children and 2.21 hours per day (95% CI, 1.23 to 3.20 hours per day) longer among Hispanic all races children. Children of women with a master’s degree or higher had 1.85 hours per day (95% CI, −2.88 to 0.82 hours per day) less total screen time, 0.49 hours per day (95% CI, −0.85 to −0.14 hours per day) less educational screen time, and 1.27 hours per day (95% CI, −2.21 to −0.33 hours per day) less recreational screen time compared with children of women with some college, no degree, or lower (Table 3).

In analyses stratified by cohort, we found the largest increase in total screen time occurred in the Colorado cohort and the smallest increase occurred in the California cohort (eTable 5 in Supplement 1). Higher levels of prepandemic screen time were negatively associated with change in total, educational, and recreational screen time between baseline and both pandemic time points (eTables 6 and 7 in Supplement 1). For example, the change in total screen time was −0.51 hours lower per additional hour of screen time prepandemic (95% CI, −0.65 to −0.37 hours). Older children had larger changes in total and recreational screen time at both pandemic time points. Maternal education was not associated with changes to screen time in the first pandemic period; however, in the second pandemic period, children of mothers who had a bachelor’s degree had higher screen time increases than children of mothers who did not have a college degree.

## Discussion

This longitudinal cohort study among children aged 4 to 12 years across the US found increases in total screen time, as well as in recreational and educational screen time, from before to during the COVID-19 pandemic that persisted as the pandemic progressed. The amount of screen time that was engaged in together with a caregiver increased during the pandemic for TV watching, educational, and computer games but decreased for video games. There was also an increase in the percentage of children with a social media account. Older age of the child prepandemic was associated with a greater increase in screen time during the pandemic.

Our findings of an increase in screen time among children during the COVID-19 pandemic are consistent with prior studies largely conducted outside the US. A recent systematic review^2^ found increases in screen time from before to during the initial COVID-19 lockdown among children and adolescents; however, the studies were largely cross-sectional. Only 8 studies had prospective data collection, studies to date lacked information on types of screen time,^2^ and none assessed whether the observed increases persisted as pandemic progressed. Of the 2 studies^5,13^ conducted in the US, one had only qualitative data and did not assess the size of possible increases^13^ and the other was conducted among a population of children^5^ with autism spectrum disorder, which might not be generalizable. We found that screen time increases persisted more than 1 year into the pandemic when many restrictions were lifted; this suggests there may need to be more clear clinical guidelines and policies to help parents and their children reduce screen time in the COVID-19 era. For example, pediatricians may need to talk to parents about the importance of implementing the American Academy of Pediatrics Family Media Plan tool,^14^ which helps families set media use priorities and limits that may have been relaxed during the early stages of the COVID-19 pandemic. We found that throughout the study period, screen time levels were higher among children of Black race or Hispanic ethnicity, older children, and children whose mothers had less than a college education. A prepandemic study^15^ of screen time usage among approximately 600 young children found that screen time was higher among racial and ethnic minoritized populations and among children of parents with lower socioeconomic status. It has been hypothesized that these disparities may be associated with lack of access to structured activities for children outside of school.^16,17,18^ The early COVID-19 pandemic led to unprecedented increases in unstructured time for all children and exacerbated social and economic disparities,^19,20^ and our results suggest that children from minoritized groups experienced larger increases in screen time. The observed racial, ethnic, and socioeconomic disparities in screen time are likely associated with structural barriers or lack of opportunities and poverty-related factors that may have increased reliance on screen time. The observed disparities in screen time highlight the need for policy level interventions to improve access to essential resources and opportunities across racial and ethnic groups.

We lacked information on the effects of screen time on health outcomes such as obesity and mental health in this study; however, the Adolescent Brain Cognitive Development Study^21^ of 12 000 children found each additional hour of total screen time per day was prospectively associated with a 0.22 higher body mass index percentile (95% CI, 0.10-0.34 percentile) at 1-year follow-up, after adjusting for covariates. In addition, a recent cross-sectional study^22^ found that more physical activity and less screen time were associated with better mental health for children. This suggests a potential strategy for mitigating the adverse effects of the increased screen time during the pandemic. In addition, we found that 11.2% of children had a social media account in the latter pandemic period compared with 4.4% before the pandemic. This is concerning, because all children in our study were younger than 13 years, which is the minimum age required for social media accounts (eg, Instagram and TikTok). Recent studies show that social media may be harmful, especially for girls, and, in a nationally representative study,^23^ the use of social media was associated with clinically significant levels of depressive symptoms and self-harm, whereas other type of media use showed no associations. It will be important to understand the long-term health impacts of the increased screen time and whether they vary by type of screen time.

### Strengths and Limitations

This study had several strengths. The ECHO Program was uniquely able to prospectively measure changes in screen time from the prepandemic period to during the pandemic using a common protocol for assessing children’s media use across time and cohorts. The study included sites from 3 different US states with varying degrees of COVID-19 precautions; thus, our sample is geographically diverse. Limitations of this study include that the survey did not assess whether children were attending remote schooling at the time the survey was conducted and whether screen time was used for remote schooling because that was a rare occurrence before the pandemic. This may have impacted how parents reported educational screen time during the pandemic period. In addition, parent-reported screen time, which correlates with objectively measured media use, often overestimates or underestimates true usage; thus, parents may have inaccurately reported their child’s media use.^24^ By including multiple cohorts across the US rather than a single cohort, we were better able to examine screen time across diverse geographic regions, sociodemographic subgroups, and child life stages. However, we acknowledge that this substudy nested in ECHO only included 3 of 69 ECHO cohorts. Therefore, these findings may not be representative of the entire US population. In addition, the 3 data collection time periods occurred at different times during the year, and changes in routines due to school calendars may have impacted screen time use. We were also underpowered to examine specific types of screen time and how associations varied by race and ethnicity and by geographic region of the various study sites.

## Conclusions

In summary, the findings of this cohort study suggest that in the US, screen time increased 1.75 hours per day during the early pandemic and remained 1.11 hours per day higher than the prepandemic period as the pandemic progressed. The increased screen time was largely associated with increases in recreational screen time, and for most types of screen time, the percentage of time that included engagement with a caretaker increased during the pandemic. Families may need support to re-establish healthy screen time usage and healthy behaviors as the pandemic continues. Pediatricians may need to provide more guidance and strategies regarding healthy screen time usage for children’s health and well-being. Future studies are needed to determine the association of increased screen time with longer term obesity and mental health outcomes in children.

## Notes

Supplement 1. **eTable 1.** Description of Cohorts Included in Analytic Sample **eTable 2.** Comparison of Participants Who Completed the Survey Versus Those Consented but Who Did Not Complete the Survey (n=317) **eTable 3.** Characteristics of Study Sample, Stratified by Cohort **eTable 4.** Prepandemic Screen Time by Cohort **eTable 5.** Estimated Associations Between Total Screen Time and Pandemic Periods, Stratified by Cohort **eTable 6.** Estimated Change in Total Screen Time, Educational Screen Time, and Recreational Screen Time Between the First Pandemic and the Prepandemic Time Period **eTable 7.** Estimated Change in Total Screen Time, Educational Screen Time, and Recreational Screen Time Between the Second Pandemic and the Prepandemic Time Period

Supplement 2.
Environmental Influences on Child Health Outcomes Program Group Members

Supplement 3.
Data Sharing Statement
