# Supplementary material for: Trends in Screen Time Use Among Children During the COVID-19 Pandemic, July 2019 Through August 2021
Source: JAMA Netw Open. 2023 Feb 15;6(2):e2256157. doi: 10.1001/jamanetworkopen.2022.56157 (PMC9932850; doi:10.1001/jamanetworkopen.2022.56157)
Supplement: Supplement 1. — eTable 1. Description of Cohorts Included in Analytic Sample eTable 2. Comparison of Participants Who Completed the Survey Versus Those Consented but Who Did Not Complete the Survey (n=317) eTable 3. Characteristics of Study Sample, Stratified by Cohort eTable 4. Prepandemic Screen Time by Cohort eTable 5. Estimated Associations Between Total Screen Time and Pandemic Periods, Stratified by Cohort eTable 6. Estimated Change in Total Screen Time, Educational Screen Time, and Recreational Screen Time Between the First Pandemic and the Prepandemic Time Period eTable 7. Estimated Change in Total Screen Time, Educational Screen Time, and Recreational Screen Time Between the Second Pandemic and the Prepandemic Time Period [file jamanetwopen-e2256157-s001.pdf]

Supplemental Online Content

Hedderson MM, Bekelman TA, Li M, et al; Environmental Influences on Child Health Outcomes Program. Trends in screen time use among children during the COVID-19 pandemic, July 2019 through August 2021. *JAMA Netw Open*. 2023;6(2):e2256157. doi:10.1001/jamanetworkopen.2022.56157

eTable 1. Description of Cohorts Included in Analytic Sample

eTable 2. Comparison of Participants Who Completed the Survey Versus Those Consented but Who Did Not Complete the Survey (n=317)

eTable 3. Characteristics of Study Sample, Stratified by Cohort

eTable 4. Prepandemic Screen Time by Cohort

eTable 5. Estimated Associations Between Total Screen Time and Pandemic Periods, Stratified by Cohort

eTable 6. Estimated Change in Total Screen Time, Educational Screen Time, and Recreational Screen Time Between the First Pandemic and the Prepandemic Time Period

eTable 7. Estimated Change in Total Screen Time, Educational Screen Time, and Recreational Screen Time Between the Second Pandemic and the Prepandemic Time Period

This supplemental material has been provided by the authors to give readers additional information about their work.

© 2023 Hedderson MM et al. *JAMA Network Open.*

eTable 1. Description of Cohorts Included in Analytic Sample

Award	Cohort Name<br><br>Citation	Recruitment Life Stage	Type of Cohort	Target Population	Primary ECHO Outcome Area(s)
The Early Life Exposome and Childhood Health – The Colorado Healthy Start 3 Cohort Study	Healthy Start[1]	Prenatal	Clinic/hospital-based; Community-based	General population<br><br>Healthy pregnant women without prior adverse birth outcomes	Obesity
Environmental Influences on Child Health Outcomes in the Northern Plains Safe Passage Study Cohort	Safe Passage Study[2] (PASS)	Prenatal	Clinic/hospital-based	Rural and American Indian pregnant women with singletons or twins living in communities with historically high prenatal alcohol use	Pre-, Peri-, Postnatal<br><br>Airways<br><br>Neurodevelopment
Early Life Exposure to Endocrine Disrupting Chemicals and Child Growth; Adiposity; and Neurodevelopment	Pregnancy Environment and Lifestyle Study[3] (PETALS)	Prenatal	Clinic/hospital-based	General population<br><br>Healthy pregnant women with singletons	Pre-, Peri-, Postnatal
	Kaiser Permanente Research Bank[4] (KPRB)	Prenatal	Clinic/hospital-based	General population<br><br>Healthy pregnant women	Pre-, Peri-, Postnatal

© 2023 Hedderson MM et al. *JAMA Network Open.*

eTable 2. Comparison of Participants Who Completed the Survey Versus Those Consented but Who Did Not Complete the Survey (n=317)

	Completed survey during pandemic (n=228)	Did not complete survey during pandemic (n=89)
Race & Ethnicity, n(%)		
Non-Hispanic White	108 (47.4%)	29 (31.5%)
Non-Hispanic Black	18 (7.9%)	7 (7.9%)
Non-Hispanic other	37 (16.2%)	25 (28.1%)
Hispanic, all races	65 (28.5%)	28 (31.5%)
Maternal Education, n (%)		
Some college, no degree or lower	71 (31.1%)	47 (52.8%)
Bachelor’s degree or higher	157 (68.8%)	39 (42.7%)
Missing	0	3 (3.4%)

© 2023 Hedderson MM et al. *JAMA Network Open.*

eTable 3. Characteristics of Study Sample, Stratified by Cohort

Cohort	All Cohorts	Colorado	South Dakota	California
**N**	**228**	**74**	**39**	**115**
**Female sex, n (%)**	100 (43.9%)	36 (48.6%)	19 (48.7%)	45 (39.1%)
**Baseline child age in years, mean (SD)**	7.0 (2.7)	8.7(0.5)	11.2 (0.2)	4.47 (0.2)
**Baseline Age group, yrs old, n (%)**				
4 to <5	115 (50.4%)	0 (0%)	0 (0%)	115 (100%)
5 to <9	50 (21.9%)	50 (67.6%)	0 (0%)	0 (0%)
≥9	63 (27.6%)	24 (32.4%)	39 (100%)	0 (0%)
**Race & Ethnicity, n (%)** [1]				
Non-Hispanic White	108 (47.4%)	40 (54.1%)	31 (79.5%)	37 (32.2%)
Non-Hispanic Black	18 (7.9%)	13 (17.6%)	0 (0%)	5 (4.3%)
Non-Hispanic other	37 (16.2%)	7 (9.5%)	<8 (<18%)	23 (20.0%)
Hispanic all races	65 (28.5%)	14 (18.9%)	<5 (<3%)	50 (43.5%)
**Maternal Education, n (%)** [2]				
Some college, no degree or lower	71 (31.1%)	18 (24.3%)	9 (23.1%)	44 (38.3%)
Bachelor's degree	80 (35.1%)	20 (27.0%)	21 (53.8%)	39 (33.9%)
Master's degree or higher	77 (33.8%)	36 (48.6%)	9 (23.1%)	32 (27.8%)
**Annual household income , n (%)**				
<$30,000	11 (4.8%)	6 (8.1%)	<5	<5
$30,000- $49,999	24 (10.5%)	11 (14.9%)	5 (12.8%)	10 (8.7%)
$50,000- $74,999	32 (14.0%)	11 (14.9%)	<5	18 (15.7%)
$75,000-$99,999	32 (14.0%)	8 (10.8%)	6 (15.4%)	18 (15.7%)
>=$100,000	124 (54.4%)	<40 (<55%)	22 (56.4%)	66 (57.4%)
Missing	5 (2.2%)	<5	<5	<5
**Number of siblings in household, n (%)**				
0	15 (6.6%)	<10	<5	9 (7.8%)
1	101 (44.3%)	30 (40.5%)	13 (33.3%)	58 (50.4%)
2	65 (28.5%)	22 (29.7%)	14 (35.9%)	29 (25.2%)
3	29 (12.7%)	9 (12.2%)	8 (20.5%)	12 (10.4%)
≥4	13 (5.7%)	<5	<5	7 (6.1%)
Missing	5 (2.2%)	<5	<5	0 (0%)

© 2023 Hedderson MM et al. *JAMA Network Open.*

**How COVID 19 affected maternal job (% yes)**				
Moved to working remotely or from home	89 (39.0%)	28 (37.8%)	11 (28.2%)	50 (43.5%)
Missing	<5	<5	0 (0%)	<5
Did not have a paying job before	23 (10.1%)	9 (12.2%)	<5	12 (10.4%)
Missing	<5	<5	0 (0%)	<5
Lost the job permanently/ temporarily/ Reduced work hours	56 (24.6%)	18 (24.3%)	6 (15.4%)	32 (27.8%)
Missing	<5	<5	0 (0%)	<5
Got a new job/ Increased work hours	29 (12.7%)	8 (10.8%)	<5	18 (15.7%)
Missing	<5	<5	0 (0%)	<5

[1] Non-Hispanic other includes 13 Asian, 7 American Indian or Alaska Native, and 17 Multiple race children.

[2] Some college, no degree or lower including associate degree, trade school, high school degree, GED or equivalent, and less than high school. Master's degree or higher also include (professional) doctoral degree.

[3] Children were included in this sample if they had at least one of the same screen time outcomes for both pre-pandemic (July 1, 2019- March 15, 2020) and one post-pandemic periods (December 1, 2020 – April 30, 2021 or May 1, 2021 – August 31, 2021).

© 2023 Hedderson MM et al. *JAMA Network Open.*

eTable 4. Prepandemic Screen Time by Cohort

Outcomes	Colorado	South Dakota	California
**N**	**(N= 74)**	**(N= 39)**	**(N=115)**
**Screen time, hrs/day, mean (SD) median [IQR]**			
**Total duration**	5.2 (4.8)	4.9 (3.2)	3.6 (3.3)
	3.4 [1.6, 7.6]	4.3 [2.7, 5.6]	2.8 [1.5, 4.4]
**Weekday duration**	4.7 (5.0)	4.3 (3.4)	3.5 (3.8)
	2.6 [1.0, 6.7]	3.5 [2.1, 4.5]	2.5 [1.5, 4.0]
**Weekend duration**	6.2 (4.6)	6.4 (3.4)	3.9 (2.8)
	4.0 [3.0, 8.9]	6.0 [4.0, 8.0]	3.3 [2.0, 5.0]
**Educational duration[b]**	0.69 (1.7)	0.62 (0.91)	0.25 (0.60)
	0 [0, 0.56]	0.25 [0, 0.83]	0 [0, 0.26]
**Recreational duration[c]**	4.7 (4.3)	4.3 (2.6)	3.4 (3.2)
	3.0 [1.6, 6.4]	3.6 [2.4, 5.2]	2.6 [1.5, 4.0]
Watching TV[d]	2.1 (1.9)	1.9 (1.3)	2.2 (2.1)
	1.6 [0.86, 2.8]	1.6 [1.1, 2.5]	1.6 [1.0, 2.7]
Playing Games[e]	1.9 (2.8)	1.6 (1.3)	0.86 (1.7)
	1.0 [0.30, 1.9]	1.3 [0.63, 2.3]	0.36 [0, 0.97]
Video Chatting	0.12 (0.45)	0.087 (0.25)	0.079 (0.17)
	0 [0, 0]	0 [0, 0]	0 [0, 0.080]
Browsing Website	0.13 (0.35)	0.25 (0.47)	0.019 (0.20)
	0 [0, 0]	0 [0, 0.40]	0 [0, 0]
Others[f]	0.74 (1.6)	0.44 (0.74)	0.22 (0.74)
	0.18 [0, 0.90]	0.12 [0, 0.55]	0 [0, 0.13]
**Social media account**			
Yes	<5	<10	0 (0%)
No	70 (94.6%)	29 (74.4%)	115 (100%)
missing	<5	<5	0 (0%)
**Watch TV with caregivers**			
Some of the time/Most of the time	8 (10.8%)	5 (12.8%)	20 (17.4%)
Never/Hardly ever	66 (89.2%)	34 (87.2%)	95 (82.6%)
missing	0 (0%)	0 (0%)	0 (0%)
**Play video game with caregivers**			
Some of the time/Most of the time	46 (62.2%)	25 (64.1%)	94 (81.7%)
Never/Hardly ever	<30	<15	<25
missing	<5	<5	<5

© 2023 Hedderson MM et al. *JAMA Network Open.*

**Play computer game with caregivers**			
Some of the time/Most of the time	52 (70.3%)	30 (76.9%)	101 (87.8%)
Never/Hardly ever	<20	<10	<15
missing	<5	<5	<5
**Play games on an education game device with caregivers**			
Some of the time/Most of the time	59 (79.7%)	34 (87.2%)	82 (71.3%)
Never/Hardly ever	9 (12.2%)	<5	32 (27.8%)
missing	6 (8.1%)	<5	<5
**Play games or use apps on a smartphone or tablet with caregivers**			
Some of the time/Most of the time	39 (52.7%)	24 (61.5%)	55 (47.8%)
Never/Hardly ever	<35	15 (38.5%)	<60
missing	<5	0 (0%)	<5
**Video-chat with caregivers**			
Some of the time/Most of the time	52 (70.3%)	32 (82.1%)	77 (67.0%)
Never/Hardly ever	<20	<10	<40
missing	<5	<5	<5

[a]July 1, 2019- March 15, 2020

[b]Includes “Playing games on an education game device (such as Leapster / LeapPad, LeapFrog Epic, Playtime Pad, or V-Tech device - V-Smile, Mobigo, or Innotab)” and “Doing homework on a computer or tablet”. This is a weighted average of weekday and weekend duration.

[c]Includes “Watching TV shows, DVDs, or videos”, “Playing video games”, “Playing computer games”, “Playing games or using apps on a smartphone or tablet”, “Video Chatting”, “Browsing websites”, “Doing anything else on a computer”, and “Doing anything else on a smartphone or tablet”. This is a weighted average of weekday and weekend duration.

[d]Watching TV shows, DVDs, or videos (include time spent watching on a TV set, computer, smartphone, and tablet; this includes watching shows that may have been previously recorded). This is a weighted average of weekday and weekend duration.

[e]Includes “Playing video games”, “Playing computer games”, and “Playing games or using apps on a smartphone or tablet”. This is a weighted average of weekday and weekend duration.

[f]Includes “Doing anything else on a computer (such as looking at pictures, looking up things, social networking, emailing, shopping, text messaging)” and “Doing anything else on a smartphone or tablet (such as taking or looking at pictures, looking up things, social networking, emailing, text messaging, or using other types of apps not already covered)”. This is a weighted average of weekday and weekend duration.

© 2023 Hedderson MM et al. *JAMA Network Open.*

eTable 5. Estimated Associations Between Total Screen Time and Pandemic Periods, Stratified by Cohort

	Colorado	South Dakota	California
**N of Observations**	187	109	322
**Time point**			
Pandemic 1	3.41 (1.25, 5.56)	3.84 (-2.38, 10.06)	1.43 (-1.08, 3.95)
Pandemic 2	2.86 (0.14, 5.59)	4.60 (-3.16, 12.37)	0.62 (-2.45, 3.70)
**Age, yrs old**	-0.46 (-1.98, 1.06)	-1.12 (-6.14, 3.90)	0.52 (-1.53, 2.57)
**Male**	-0.30 (-1.74, 1.13)	-0.99 (-2.92, 0.94)	0.02 (-1.02, 1.05)
**Number of siblings**	0.27 (-0.36, 0.90)	-0.68 (-1.89, 0.53)	0.11 (-0.38, 0.60)
**Race & Ethnicity**			
Hispanic all races, n (%)	4.56 (2.65, 6.48)	0.28 (-6.95, 7.52)	1.78 (0.58, 2.99)
Non-Hispanic Black, n (%)	7.04 (5.10, 8.97)	Not estimable	1.71 (-0.91, 4.33)
Non-Hispanic other , n (%)	3.63 (0.97, 6.29)	0.77 (-2.00, 3.53)	1.34 (-0.08, 2.75)
Non-Hispanic White, n (%)	REF	REF	REF
**Maternal Education, n (%)**			
Some college, no degree or lower	REF	REF	REF
Bachelor's degree	-3.15 (-5.16, 1.14)	0.18 (-2.44, 2.80)	-1.31 (-2.54, -0.09)
Master's degree or higher	-2.37 (-4.16, -0.57)	-1.42 (-4.32, 1.47)	-1.93 (-3.33, -0.54)

[1] Beta coefficients from linear mixed effects models that include cohort and child as random intercepts to adjust for the within cohort and within child correlations. Each screentime outcome represents the weighted average of weekday and weekend parent-reported screentime. Maximum Likelihood method was used to fit model. P-values were given from LMEM t-test. The analysis was conducted using the R package "lme4".

[3] The reference group is the pre-pandemic period: July 1, 2019- March 15, 2020. Pandemic data collection period 1 is December 1, 2020 – April 30, 2021, and pandemic period 2 is May 1, 2021 – August 31, 2021.

© 2023 Hedderson MM et al. *JAMA Network Open.*

eTable 6. Estimated Change in Total Screen Time, Educational Screen Time, and Recreational Screen Time Between the First Pandemic and the Prepandemic Time Period

	Total screen time (hours/day)[a]	Educational screen time (hours/day)[a]	Recreational screen time (hours/day)[a]
**N of Observations**	211	211	211
**Pre-pandemic screen time[b]**	-0.51 (-0.65, -0.37)	-0.82 (-1.04, -0.59)	-0.44 (-0.58, -0.31)
**Baseline age**	0.32 (0.12, 0.52)	0.09 (-0.02, 0.20)	0.29 (0.12, 0.46)
**Male**	-0.67 (-1.63, 0.28)	-0.24 (-0.74, 0.25)	-0.84 (-1.67, -0.01)
**Number of siblings**	0.45 (-0.01, 0.90)	0.14 (-0.09, 0.38)	0.39 (-0.003, 0.79)
**Race & Ethnicity**			
Hispanic all races	1.82 (0.52, 3.12)	0.93 (0.28, 1.59)	1.01 (-0.11, 2.14)
Non-Hispanic Black	3.94 (1.95, 5.93)	1.53 (0.53, 2.54)	2.85 (1.13, 4.57)
Non-Hispanic Other	2.16 (0.77, 3.54)	1.57 (0.86, 2.29)	1.06 (-0.15, 2.26)
Non-Hispanic White	REF	REF	REF
**Maternal Education**			
Some college, no degree or lower	REF	REF	REF
Bachelor's degree	-0.43 (-1.62, 0.76)	-0.24 (-0.85, 0.37)	-0.23 (-1.26, 0.81)
Master's degree or higher	-0.74 (-2.00, 0.53)	-0.66 (-1.30, -0.02)	-0.01 (-1.11, 1.08)

[a]Beta coefficients from linear mixed effects models that include cohort as a random intercept to adjust for the within cohort correlations. Each change in screentime outcome represents the difference in the weighted average of weekday and weekend parent-reported screentime between pandemic time period 1 and the pre-pandemic time period. The maximum Likelihood method was used to fit model. P-values were given from LMEM t-test. The analysis was conducted using the R package "lme4".

[b]The pre-pandemic period is July 1, 2019- March 15, 2020. Pandemic data collection period 1 is December 1, 2020 – April 30, 2021.

© 2023 Hedderson MM et al. *JAMA Network Open.*

eTable 7. Estimated Change in Total Screen Time, Educational Screen Time, and Recreational Screen Time Between the Second Pandemic and the Prepandemic Time Period

	**Total screen time[a] (hours/day)**	**Educational screen time[a] (hours/day)**	**Recreational screen time[a] (hours/day)**
**N of Observations**	179	179	179
**Pre-pandemic screen time[b]**	-0.55 (-0.71, -0.38)	-0.87 (-1.05, -0.69)	-0.49 (-0.65, -0.32)
**Baseline age**	0.46 (0.24, 0.69)	0.07 (-0.02, 0.15)	0.47 (0.27, 0.67)
**Male**	0.35 (-0.73, 1.42)	0.15 (-0.28, 0.58)	0.22 (-0.75, 1.19)
**Number of siblings**	-0.05 (-0.57, 0.46)	-0.02 (-0.22, 0.19)	-0.04 (-0.51, 0.42)
**Race & Ethnicity**			
Hispanic all races	1.65 (0.17, 3.14)	0.52 (-0.05, 1.10)	1.25 (-0.10, 2.59)
Non-Hispanic Black	0.57 (-2.22, 3.37)	-0.55 (-1.58, 0.48)	0.82 (-1.72, 3.35)
Non-Hispanic other	1.32 (-0.25, 2.89)	0.93 (0.31, 1.55)	0.32 (-1.10, 1.74)
Non-Hispanic White	REF	REF	REF
**Maternal Education**			
Some college, no degree or lower	REF	REF	REF
Bachelor's degree	-1.90 (-3.31, -0.48)	-0.64 (-1.19, -0.08)	-1.52 (-2.80, -0.23)
Master's degree or higher	-0.92 (-2.35, 0.51)	-0.59 (-1.15, -0.04)	-0.71 (-2.01, 0.59)

[a]Beta coefficients from linear mixed effects models that include cohort as a random intercept to adjust for the within cohort correlations. Each change in screentime outcome represents the difference in the weighted average of weekday and weekend parent-reported screentime between pandemic time period 2 and the pre-pandemic time period. The maximum Likelihood method was used to fit model. P-values were given from LMEM t-test. The analysis was conducted using the R package "lme4".

[b]The pre-pandemic period is July 1, 2019- March 15, 2020. Pandemic data collection period 1 is December 1, 2020 – April 30, 2021, and pandemic period 2 is May 1, 2021 – August 31, 2021.

© 2023 Hedderson MM et al. *JAMA Network Open.*
