# Supplementary material for: Trends in Screen Time Use Among Children During the COVID-19 Pandemic, July 2019 Through August 2021
Source: JAMA Netw Open. 2023 Feb 15;6(2):e2256157. doi: 10.1001/jamanetworkopen.2022.56157 (PMC9932850; doi:10.1001/jamanetworkopen.2022.56157)
Supplement: Supplement 2. — Environmental Influences on Child Health Outcomes Program Group Members [file jamanetwopen-e2256157-s002.pdf]

Supplemental Online Content: Nonauthor Collaborators
*First name, last name, and suffix (if applicable) are required and will appear in PubMed.

***Group Name(s): Environmental Influences on Child Health Outcomes Program**							
***First Name and Middle Initial(s)**	***Last Name**	***Suffix (eg, Jr, III)**	Academic Degrees	Institution	Location (city, state/province, country)	Role or Contribution, eg, chair, principal investigator	Group (if more than 1 Group listed in the byline) and/or Subgroup (eg, Steering Committee)
Brian P	Smith		MD	Duke Clinical Research Institute	Durham, North Carolina, USA	ECHO Coordinating Center Principal Investigator	ECHO Coordinating Center U2COD023375
Kristen L	Newby		MD	Duke Clinical Research Institute	Durham, North Carolina, USA	ECHO Coordinating Center Principal Investigator	ECHO Coordinating Center U2COD023375
Lisa P	Jacobson		PhD	Johns Hopkins University	Baltimore, Maryland, USA	ECHO Data Analysis Center Principal Investigator	ECHO Data Analysis Center U24D023382
Corette B	Parker		PhD	Research Triangle Park Institute	Durham, North Carolina, USA	ECHO Data Analysis Center Principal Investigator	ECHO Data Analysis Center U24D023382
Richard C	Gershon		PhD	Northwestern University School of Medicine	Evanston, Illinois, USA	ECHO Person Reported Outcome Core Principal Investigator	ECHO Person Reported Outcome Core U24OD023319
David	Cella		PhD	Northwestern University School of Medicine	Evanston, Illinois, USA	ECHO Person Reported Outcome Core Principal Investigator	ECHO Person Reported Outcome Core U24OD023319
Akram N	Alshawabkeh		PhD	Northeastern University	Boston, Massachusetts, USA	ECHO Cohort Principal Investigator	ECHO Cohort UH3OD023251
Judy L	Aschner		MD	Albert Einstein College of Medicine	Bronx, New York, USA	ECHO Cohort Principal Investigator	ECHO Cohort UH3OD023320
Stephanie L	Merhar		MD	Cincinnati Children's Hospital Medical Center	Cincinnati, Ohio, USA	ECHO Cohort Principal Investigator	ECHO Cohort UH3OD023320
Gloria S	Pryhuber		MD	University of Rochester Medical Center	Rochester, NY, USA	ECHO Cohort Principal Investigator	ECHO Cohort UH3OD023320

Supplemental Online Content: Nonauthor Collaborators
*First name, last name, and suffix (if applicable) are required and will appear in PubMed.

*First Name and Middle Initial(s)	*Last Name	*Suffix (eg, Jr, III)	Academic Degrees	Institution	Location (city, state/province, country)	Role or Contribution, eg, chair, principal investigator	Group (if more than 1 Group listed in the byline) and/or Subgroup (eg, Steering Committee)
Paul E	Moore		MD	Vanderbilt Children's Hospital	Nashville, TN, USA	ECHO Cohort Principal Investigator	ECHO Cohort UH3OD023320
Andrea L	Lampland		MD	Children's Hospital and Clinic	Minneapolis, MN, USA	ECHO Cohort Principal Investigator	ECHO Cohort UH3OD023320
Rajan	Wadhawan		MD	Florida Hospital for Children	Orlando, FL, USA	ECHO Cohort Principal Investigator	ECHO Cohort UH3OD023320
Carol L	Wagner		MD	Medical University of South Carolina	Charleston, SC, USA	ECHO Cohort Principal Investigator	ECHO Cohort UH3OD023320
Mark L	Hudak		MD	University of Florida College of Medicine	Jacksonville, FL, USA	ECHO Cohort Principal Investigator	ECHO Cohort UH3OD023320
Dennis E	Mayock		MD	University of Washington	Seattle, WA, USA	ECHO Cohort Principal Investigator	ECHO Cohort UH3OD023320
Lisa K	Washburn		MD	Wake Forest University School of Medicine	Winston Salem, NC	ECHO Cohort Principal Investigator	ECHO Cohort UH3OD023320
Lisa A	Croen		PhD	Kaiser Permanente Northern California Division of Research	Oakland, CA, USA	ECHO Cohort Principal Investigator	ECHO Cohort UH3OD023289
Caherine J	Karr		MD	University of Washington, Department of Environmental and Occupational Health Sciences	Seattle, WA, USA	ECHO Cohort Principal Investigator	ECHO Cohort UH3OD023271
Alex	Mason		PhD	University of Tennessee Health Science Center	Memphis, TN, USA	ECHO Cohort Principal Investigator	ECHO Cohort UH3OD023271
Barry M	Lester		PhD	Women & Infants Hospital of Rhode Island	Providence RI, USA	ECHO Cohort Principal Investigator	ECHO Cohort UH3OD023347
Brian S	Carter		MD	Children's Mercy	Kansas City, MO, USA	ECHO Cohort Principal Investigator	ECHO Cohort UH3OD023347
Carmen J	Marsit		PhD	Emory University	Atlanta, GA, USA	ECHO Cohort Principal Investigator	ECHO Cohort UH3OD023347
Steven L	Pastyrnak		PhD	Helen DeVos Children's Hospital	Grand Rapids, MI, USA	ECHO Cohort Principal Investigator	ECHO Cohort UH3OD023347
Charles	Neal		MD	Kapiolani Medical Center for Women and Children	Providence, RI, USA	ECHO Cohort Principal Investigator	ECHO Cohort UH3OD023347

Supplemental Online Content: Nonauthor Collaborators
*First name, last name, and suffix (if applicable) are required and will appear in PubMed.

***First Name and Middle Initial(s)**	***Last Name**	***Suffix (eg, Jr, III)**	Academic Degrees	Institution	Location (city, state/province, country)	Role or Contribution, eg, chair, principal investigator	Group (if more than 1 Group listed in the byline) and/or Subgroup (eg, Steering Committee)
Lynne M	Smith		MD	Los Angeles Biomedical Research Institute at Harbour-UCLA Medical Center	Los Angeles CA, USA	ECHO Cohort Principal Investigator	ECHO Cohort UH3OD023347
Jennifer B	Helderman		MD	Wake Forest University School of Medicine	Winston Salem, NC	ECHO Cohort Principal Investigator	ECHO Cohort UH3OD023347
Cindy	McEvoy		MD	Oregon Health and Science University	Portland, OR, USA	ECHO Cohort Principal Investigator	ECHO Cohort UH3OD023288
Robert S	Tepper		MD	Indiana University, Riley Hospital for Children	Indianapolis, IN, USA	ECHO Cohort Principal Investigator	ECHO Cohort UH3OD023288
Jean	Kerver		PhD	Michigan State University	East Lansing, MI, USA	ECHO Cohort Principal Investigator	ECHO Cohort UH3OD023285
Charles	Barone		MD	Henry Ford Health System	Detroit, MI, USA	ECHO Cohort Principal Investigator	ECHO Cohort UH3OD023285
Patricia	McKane		DVM	Michigan Department of Health and Human Services	Lansing, MI, USA	ECHO Cohort Principal Investigator	ECHO Cohort UH3OD023285
Nigel	Paneth		MD	Michigan State University	East Lansing, MI, USA	ECHO Cohort Principal Investigator	ECHO Cohort UH3OD023285
Michael R	Elliott		PhD	University of Michigan	Ann Arbor, MI, USA	ECHO Cohort Principal Investigator	ECHO Cohort UH3OD023285
Leonardo	Trasande		MD	New York School of Medicine	New York, NY, USA	ECHO Cohort Principal Investigator	ECHO Cohort UH3OD023305
Sheela	Sathyanarayana		MD	Seattle Children's Research Institute	Seattle, WA	ECHO Cohort Principal Investigator	ECHO Cohort UH3OD023271
Nicole	Bush		PhD	University of California, San Francisco	San Francisco CA, USA	ECHO Cohort Principal Investigator	ECHO Cohort UH3OD023271 and UH3OD023282
Ruby HN	Nguyen		PhD	University of Minnesota	Minneapolis, MN, USA	ECHO Cohort Principal Investigator	ECHO Cohort UH3OD023271 and UH3OD023282

Supplemental Online Content: Nonauthor Collaborators

*First name, last name, and suffix (if applicable) are required and will appear in PubMed.

***First Name and Middle Initial(s)**	***Last Name**	***Suffix (eg, Jr, III)**	Academic Degrees	Institution	Location (city, state/province, country)	Role or Contribution, eg, chair, principal investigator	Group (if more than 1 Group listed in the byline) and/or Subgroup (eg, Steering Committee)
Emily S	Barrett		PhD	University of Rochester Medical Center	Rochester, NY, USA	ECHO Cohort Principal Investigator	ECHO Cohort UH3OD023271 and UH3OD023282
