# Supplementary material for: Trends in Screen Time Use Among Children During the COVID-19 Pandemic, July 2019 Through August 2021
Source: JAMA Netw Open. 2023 Feb 15;6(2):e2256157. doi: 10.1001/jamanetworkopen.2022.56157 (PMC9932850; doi:10.1001/jamanetworkopen.2022.56157)
Supplement: Supplement 3. — Data Sharing Statement [file jamanetwopen-e2256157-s003.pdf]

Data Sharing Statement

Hedderson. Trends in Screen Time Use Among Children During the COVID-19 Pandemic, July 2019 Through August 2021. *JAMA Netw Open*. Published February 15, 2023. doi:10.1001/jamanetworkopen.2022.56157

Data
Data available: No

Additional Information
Explanation for why data not available: Data sharing is not permitted by our IRB.
